# Papillary Renal Cell Carcinoma Seeding along a Percutaneous Biopsy Tract

**DOI:** 10.1155/2015/925254

**Published:** 2015-07-28

**Authors:** Deanne Soares, Nariman Ahmadi, Oana Crainic, John Boulas

**Affiliations:** Royal Prince Alfred Hospital, Missenden Road, Camperdown, Sydney, NSW 2050, Australia

## Abstract

We report a case of tumour seeding caused by percutaneous biopsy of a papillary renal cell carcinoma detected on pathological assessment of the partial nephrectomy specimen in a 50-year-old male. Whilst percutaneous biopsy of renal masses is considered to be safe and can be a valuable tool in the assessment of certain renal lesions, it is not without risks. This rare complication should be taken into consideration before contemplating its use in a patient.

## 1. Introduction

The use of percutaneous biopsies is useful in the diagnosis and management of renal masses and of other abdominal organs [1, 2]. This has been found to be a safe and effective tool with a complication rate of less than 0.01% [1]. However, a potential hazard of this procedure is tumour seeding, where malignant cells are deposited along the needle tract, but this is so rare in renal cell carcinoma (RCC) that its frequent use in the assessment of indeterminate renal masses has therefore been justified [1, 3]. Here we present a case of RCC seeding along a previous percutaneous renal biopsy tract noted on histopathological assessment of a partial nephrectomy specimen.

## 2. Case Presentation

**Figure 1:**
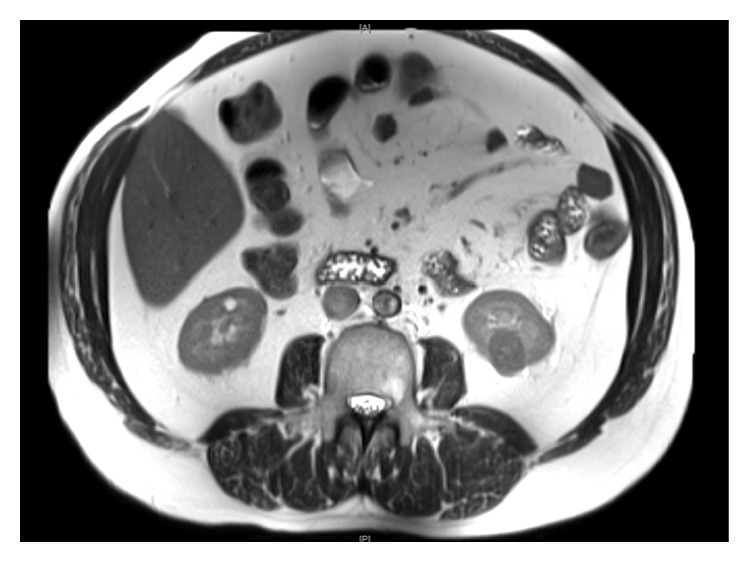
Magnetic resonance imaging (MRI) showing a 26 × 21 mm left renal lower pole mass with a low T2 signal.

A 50-year-old male was referred to our institution for review after an incidental finding of a 2.5 cm enhancing lower pole mass on the left kidney. This mass was first noted on ultrasound imaging as part of his investigations for symptoms of bloating and constipation. A computed tomography scan with intravenous contrast was then performed to further characterise this lesion, which showed a low density but mildly enhancing lesion. Magnetic resonance imaging (MRI) was performed to exclude angiomyolipoma. It revealed a 26  ×  21 mm left renal lower pole exophytic mass with a low T2 signal suggesting the possibility of a renal cell carcinoma (see Figure 1). The patient then underwent an ultrasound-guided biopsy of the mass to aid with diagnosis and assist in management options. The lesion was first accessed using a 17-gauge needle with a coaxial sheath and 2 fine needle aspiration (FNA) biopsies were performed. This was followed by core biopsies using an 18-gauge core biopsy needle also done with the coaxial sheath in place. Two passes were made to obtain 6 cores, all embedded in 2 blocks. The core sizes were 6 mm, 5 mm, 4 mm, 4 mm, 4 mm, and 3 mm in length. Pathological assessment of the FNA and core biopsy specimens confirmed the presence of a low-grade neoplasm consisting of closely packed cells with small rounded nuclei forming clusters and some mucin filled tubules. Given this finding, the patient underwent a subsequent open left partial nephrectomy 8 weeks later. The procedure involved a lower pole partial nephrectomy, with frozen section confirming clear parenchymal margins. The perinephric fat over the tumour was initially reflected off during surgery and sent separately with a marking suture placed where the fat was adherent over the tumour site. There was no tumour capsule disruption or spillage or any other complication during the procedure.

**Figure 2:**
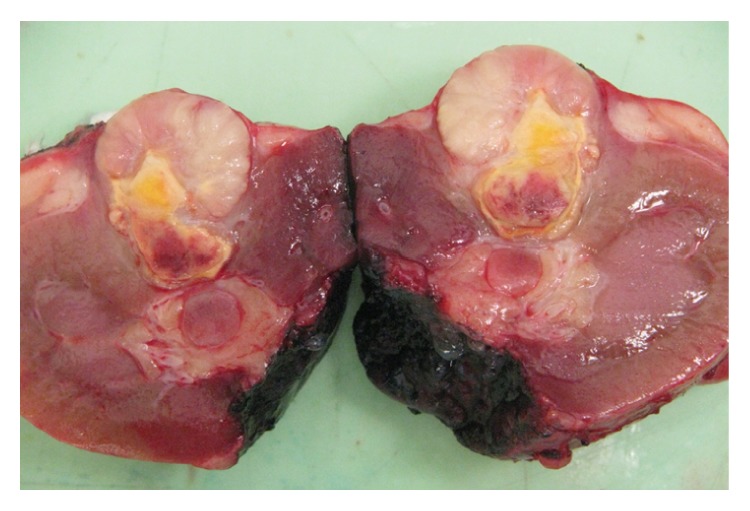
Macroscopic view of the partial nephrectomy specimen demonstrating a 23 × 20 mm well-demarcated tumour.

**Figure 3:**
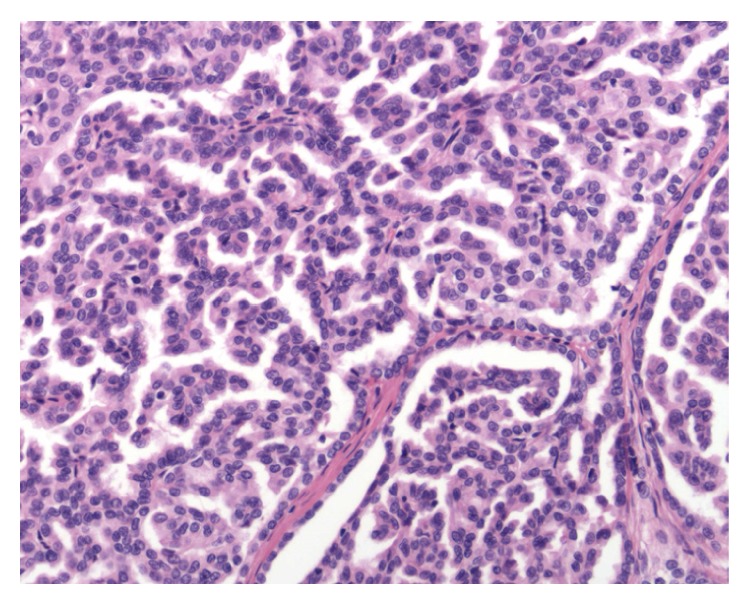
High power view of the lesion demonstrating papillary renal cell carcinoma.

**Figure 4:**
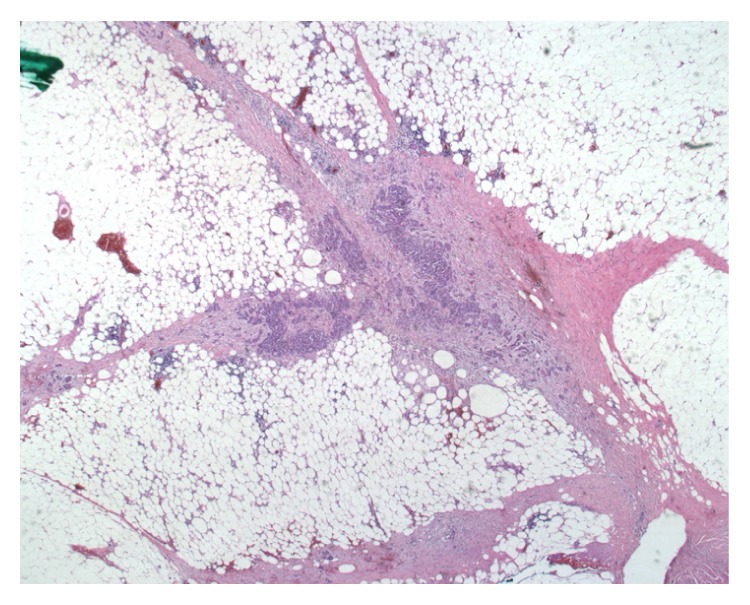
Fat overlying the tumour demonstrating papillary renal cell carcinoma seeding along the previous percutaneous biopsy tract.

**Figure 5:**
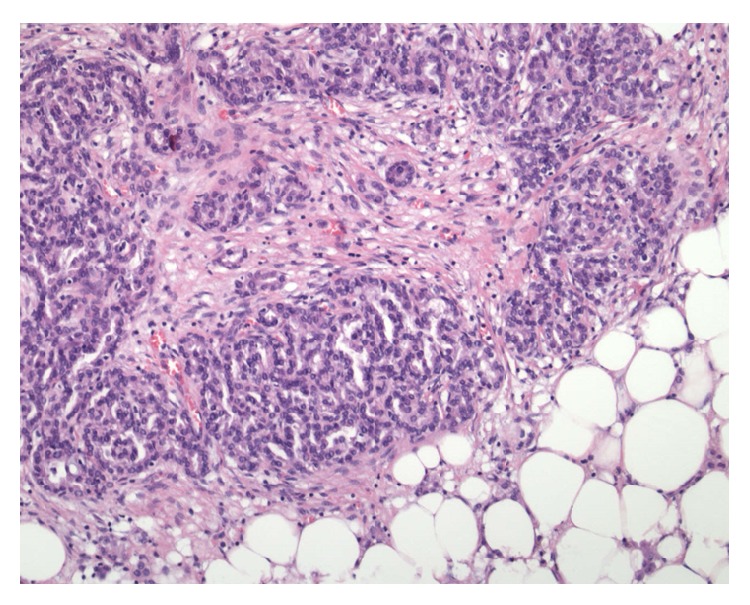
High power view of the papillary renal cell carcinoma seeding along the previous percutaneous biopsy tract.

Macroscopically, there was a lobulated grey partly necrotic, noncystic tumour measuring 23 × 20 mm (see Figure 2 showing the bisected specimen). Microscopically, the lesion was well demarcated showing a complex papillary growth pattern (see Figure 3). Immunochemically it was strongly positive for both alpha-methyl CoA racemase and CK7. Histologically this was a type 1 papillary renal cell carcinoma. During examination of the fat overlying the tumour, viable tumour was noted seeding along the previous percutaneous biopsy tract, with it growing within the fibroblastic response that marked the biopsy tract (see Figures 4 and 5). His recovery was uneventful and he was discharged on day 6 postoperatively. He remained well 1 month after his procedure. Follow-up imaging will be sought.

## 3. Discussion

Tumour seeding in a biopsy tract has been well documented in malignancies of solid organs such as in pancreatic and lung adenocarcinoma as well as hepatocellular carcinoma [3]. However, the extension of a renal cell carcinoma along a percutaneous biopsy tract is very rare with only a few reported cases on this [4–11]. Gibbons et al. noted the first case of RCC tract seeding in 1977, 20 months after aspiration of a renal lesion using an 18-gauge needle [4].  Table 1 summarises all the previously reported cases of RCC seeding along a renal percutaneous biopsy tract.

Up until 1991, there were only 5 reported cases of RCC tract seeding [4–8] and in 2013 there were further 3 cases reported [9–11]. The size of the needle used during these biopsies ranged from 14 to 25 gauges and were detected 1 month to 7 years after the initial biopsy was performed [4–11]. A review by Herts and Baker in 1995 found that needle tract seeding from percutaneous renal mass biopsy is very rare estimating the risk to be less than 0.01% [1]. Nevertheless, this is a potential risk and there have been some suggestions that this risk is increased with the use of large gauge needles, more passes with the needle, and high tumour grade [1–3]. Also, the use of coaxial biopsy technique, in which the biopsy specimen is obtained with the use of an introducer sheath, has been recommended in order to reduce the risk of tract seeding [3]. In our case, this method was used and thus highlights the fact that this does not completely eliminate the possibility of tract seeding. Other technical recommendations to prevent tumour seeding are to avoid multiple punctures of the tumour capsule, to withdraw the needle under suction, and to wipe the cores between passes [3]. In our case, both FNA and core biopsies were done as the FNA sample was insufficient and 2 passes were made with the core biopsy needle which could have contributed to the increased risk of seeding. Most of the case reports do not specifically mention the number of passes made. In the case by Mullins and Rodriguez, there were 4 passes made for FNA sampling and 2 passes made to obtain the core samples. Also, there was no use of an introducer sheath [10]. However, Sainani et al. reported the use of an introducer sheath for the 2 FNA and 1 core samples they obtained and yet found tumour seeding along the tract [11]. This suggests that there might be other contributive factors for tumour seeding along a percutaneous biopsy tract.

Seeding of tumour is when malignant cells are seen growing along the path of a tract created by a needle usually following diagnostic needling or a closed ablation procedure and are highly site specific [2, 11]. This needs to be distinguished from local recurrence, which is the development of tumour at or in close proximity to the primary tumour usually as a consequence of suboptimal treatment or microscopic deposition of tumour cells in the surrounding tissue [2, 11]. Difficulties arise in histologically differentiating the two when a local recurrence incites a desmoplastic response to mimic a healed needle track or when trying to identify a core tract on sections that may not have been cut longitudinal to the axis of the core. This leaves the delineation reliant on correlating the site of the biopsy with the radiological images and checking for tumour multifocality elsewhere in the tumour bed. In one of the aforementioned cases, Giorgadze et al. recognised the possibility that the retroperitoneal mass that they found 4 years after the biopsy could have been due to recurrence rather than true seeding, given the presence of lymphadenopathy [9]. In our case though, the presence of tumour in fat is undoubtedly secondary to needle tract seeding as it linearly follows the path of the needle (long and narrow tract with radial extension from the long axis). In addition to the cases in Table 1, another case of cutaneous seeding has also been reported after biopsy of a pulmonary metastatic deposit of RCC [12], suggesting that the grade of the tumour may also play a role in tract seeding. However, several of the cases that have reported seeding in RCC, including ours, have been low-grade papillary type which is contrary to the suggestion that high grade tumours are more likely to seed. A possible explanation of this is that lower grade tumour cells can survive longer in the blood or clot tract induced by the needle, due to its lower metabolic requirements. Further studies are needed to investigate this.

In general terms, tract seeding will relate to the amount of disruption of the tumour capsule (needle calibre and number of punctures), pressure of egress at the puncture site (e.g., cystic masses or escaping haematoma), whether tumour cells are dropped from the needle on its withdrawal (failure to maintain negative pressure and burred needle tip), and the ability of tumour cells to survive when deposited into a scar [1, 2, 13]. In the case of renal cell carcinoma, there is a potential for underrecognition of tract seeding unless the perinephric fat is carefully histologically examined and the puncture site is marked by the surgeon. Most pathologists, including those at our institution, do a very thorough examination of the tumour but with a limited sampling of the overlying fat as that is standard practice. Seeding of tumour along a needle tract though would be very difficult to find and was very likely a chance discovery in our case. This raises the question of whether tumour tract seeding is underreported. This is important in clinical practice because it has the potential to upstage a tumour from a T1 to a T2 or T4 due to extension into perinephric fat or into the abdominal wall and thus potentially affect long-term survival.

Despite mixed reports about its diagnostic accuracy, the practice of using percutaneous renal biopsies has increased recently due to technological advances in imaging and equipment used [2]. This has led to improvements in safety and decreased rates of complications further supporting its use [2]. In this case, imaging results were consistent with a papillary RCC and given the patient's life expectancy of >30 years, our recommendation was for him to have a partial nephrectomy. However, the biopsy was done to overcome patient reluctance and further strengthen our case for surgical management, which is highly invasive and not without risks. The aim of this paper is not to deter surgeons from the use of renal biopsy but to simply add another element to consider prior to its use and to make a case for improved patient selection.

This is one of only a few contemporary case reports of RCC seeding along a percutaneous biopsy tract. Whilst this complication is so rare that it does not warrant a need to cease the use of percutaneous biopsy of renal masses, it certainly highlights the possibility of tract seeding as a potential hazard. As such, certain considerations, such as appropriate patient selection, the use of correct equipment, and suitable biopsy technique, should be made to minimise the risk of this complication.

## Figures and Tables

**Table 1:** Summary of reported cases of RCC seeding along a renal percutaneous biopsy tract.

Reference (year)	Type of tumour	Needle calibre (gauge)	Interval between biopsy and seeding	Size	Description and location
Gibbons et al. (1977) [4]	RCC	18	20 months	2 cm	Firm mass inferior to the posterior part of the right 11th rib

Auvert et al. (1982) [5]	Oncocytoma	Did not mention	7 years	2 cm	Subcutaneous mass at biopsy site

Kiser et al. (1986) [6]	Papillary RCC	14	1 month	5 mm	Nodule found when dissecting Gerota's fascia off psoas muscle

Wehle and Grabstald (1986) [7]	RCC	20	Not specified	Not specified	Flank mass at biopsy site

Shenoy et al. (1991) [8]	RCC	23	12 months	1.5 cm	Subcutaneous nodule

Giorgadze et al. (2013) [9]	Papillary RCC	22 (FNA) 20 (Core)	4 years	15 cm	Left retroperitoneal mass extending posterior to the abdominal aorta with possible invasion into the psoas muscle

Mullins and Rodriguez (2013) [10]	Papillary RCC	22 (FNA) 20 (Core)	18 months	6.5 cm	Tumour invading the perirenal fat within the previous biopsy tract

Sainani et al. (2013) [11]	Papillary RCC	25 (FNA) 20 (Core)	4 years	Up to 1.2 cm	2 retroperitoneal nodules and 1 in the paraspinal musculature

## References

[1] Herts B. R., Baker M. E. (1995). The current role of percutaneous biopsy in the evaluation of renal masses. *Seminars in Urologic Oncology*.

[2] Robertson E. G., Baxter G. (2011). Tumour seeding following percutaneous needle biopsy: the real story!. *Clinical Radiology*.

[3] Volpe A., Kachura J. R., Geddie W. R. (2007). Techniques, safety and accuracy of sampling of renal tumors by fine needle aspiration and core biopsy. *The Journal of Urology*.

[4] Gibbons R. P., Bush W. H., Burnett L. L. (1977). Needle tract seeding following aspiration of renal cell carcinoma. *Journal of Urology*.

[5] Auvert J., Abbou C. C., Lavarenne V. (1982). Needle tract seeding following puncture of renal oncocytoma. *Progress in Clinical and Biological Research*.

[6] Kiser G. C., Totonchy M., Barry J. M. (1986). Needle tract seeding after percutaneous renal adenocarcinoma aspiration. *Journal of Urology*.

[7] Wehle M. J., Grabstald H. (1986). Contraindications to needle aspiration of a solid renal mass: tumor dissemination by renal needle aspiration. *Journal of Urology*.

[8] Shenoy P. D., Lakhkar B. N., Ghosh M. K., Patil U. D. (1991). Cutaneous seeding of renal carcinoma by Chiba needle aspiration biopsy. Case report. *Acta Radiologica*.

[9] Giorgadze T., Qureshi F., Aulicino M., Jacques S. M. (2013). Retroperitoneal recurrence of a stage 1 renal cell carcinoma four years following core biopsy and fine needle aspiration: possible needle tract seeding. *Diagnostic Cytopathology*.

[10] Mullins J. K., Rodriguez R. (2013). Renal cell carcinoma seeding of a percutaneous biopsy tract. *Journal of the Canadian Urological Association*.

[11] Sainani N. I., Tatli S., Anthony S. G., Shyn P. B., Tuncali K., Silverman S. G. (2013). Successful percutaneous radiologic management of renal cell carcinoma tumor seeding caused by percutaneous biopsy performed before ablation. *Journal of Vascular and Interventional Radiology*.

[12] Jilani G., Mohamed D., Wadia H. (2010). Cutaneous metastasis of renal cell carcinoma through percutaneous fine needle aspiration biopsy: case report. *Dermatology Online Journal*.

[13] Tyagi R., Dey P. (2014). Needle tract seeding: an avoidable complication. *Diagnostic Cytopathology*.

